# From guidelines to local realities: evaluation of oral rehydration therapy and zinc supplementation in Guatemala

**DOI:** 10.26633/RPSP.2017.8

**Published:** 2017-02-08

**Authors:** Rachel Hall-Clifford, Roxanne Amerson

**Affiliations:** 1 Agnes Scott College Department of Sociology and Anthropology and Department of Public Health DecaturGeorgia United States of America Agnes Scott College, Department of Sociology and Anthropology and Department of Public Health, Decatur, Georgia, United States of America.; 2 Clemson University School of Nursing CarolinClemsonSoua United States of America Clemson University, School of Nursing, Clemson, South Carolina, United States of America.

**Keywords:** Diarrhea, fluid therapy, zinc, community health workers, Guatemala, Diarrea, fluidoterapia, zinc, agentes comunitarios de salud, Guatemala

## Abstract

**Objective.:**

*Diarrhea remains a leading cause of morbidity and mortality for children in low- and middle-income countries throughout the Americas. The World Health Organization (WHO) has developed guidelines on incorporating zinc supplementation (ZS) with traditional oral rehydration therapy (ORT) in order to shorten the duration of diarrheal episodes and to reduce poor health outcomes. Guatemala adopted these guidelines in 2011, but they have not yet been fully implemented at the community level. The objectives of this study were: (1) to co-design an ORT/ZS training program for community members with local health promoters that is appropriate to the local context and (2) to understand how attitudes and behaviors of community members changed after receiving training from the study promoters*.

**Methods.:**

*In an observational study, community health promoters in rural Guatemala were trained according to WHO guidelines, and they worked collaboratively with the study team to develop a training curriculum to implement in their community. Community-based surveys, interviews, and focus group discussions were used to assess acceptability, accessibility, and availability of oral rehydration therapy and zinc supplementation*.

**Results.:**

*Use of ORT increased from 63% to 95% among community members following training by local health promoters. Satisfaction with the service offered by health promoters increased from 63% to 90% amongst community members trained by the study promoters. However, knowledge and use of zinc supplementation remained low, which was attributable to unavailability of zinc in the study community*.

**Conclusions.:**

*Use of trained community health promoters is an effective way to translate WHO guidelines to local contexts and overcome sociocultural barriers to care. However, the health system’s structure must support availability of essential medicines in order to effectively implement those guidelines*.

## RESUMEN

**Objetivo.:**

La diarrea sigue siendo una de las causas principales de morbilidad y mortalidad en los niños de países de ingresos bajos y medianos en toda la Región de las Américas. La Organización Mundial de la Salud (OMS) ha formulado directrices relativas a la incorporación de los suplementos de cinc al tratamiento tradicional de rehidratación oral (TRO) para acortar la duración de los episodios diarreicos y disminuir los resultados deficientes en materia de salud. Guatemala adoptó estas directrices en el 2011, pero todavía no se las aplica plenamente a nivel de la comunidad. Los objetivos de este estudio fueron: 1) codiseñar un programa de capacitación sobre el TRO y los suplementos de cinc dirigido a los miembros de la comunidad por intermedio de promotores locales de salud que sea apropiado para el contexto local y 2) comprender cómo las actitudes y los comportamientos de los miembros de la comunidad cambiaron después de recibir la capacitación de los promotores que formaban parte del estudio.

**Métodos.:**

Como parte de un estudio de observación, se capacitó a un grupo de promotores comunitarios de salud en zonas rurales de Guatemala sobre las directrices de la OMS, quienes trabajaron conjuntamente con el equipo del estudio para elaborar un programa de capacitación que se ejecutaría en su comunidad. Se usaron encuestas, entrevistas y debates con grupos de sondeo en las comunidades para evaluar la aceptabilidad, accesibilidad y disponibilidad del tratamiento de rehidratación oral y la administración de suplementos de cinc.

**Resultados.:**

El uso de la TRO aumentó de 63% a 95% entre los miembros de la comunidad después de la capacitación a cargo de los promotores locales de salud. La satisfacción con el servicio ofrecido por los promotores de salud aumentó de 63% a 90% entre los miembros de la comunidad capacitados por los promotores del estudio. Sin embargo, el conocimiento y uso de los suplementos de cinc siguieron siendo bajos, lo que se atribuyó a la falta de disponibilidad de cinc en la comunidad del estudio.

**Conclusiones.:**

El uso de promotores comunitarios de salud capacitados es una manera eficaz de traducir las directrices de la OMS a los contextos locales y superar los obstáculos socioculturales a la atención. Sin embargo, la estructura del sistema de salud debe apoyar la disponibilidad de los medicamentos esenciales para aplicar eficazmente estas directrices.

For many decades, around the world, diarrhea has remained the second leading cause of death in children under 5 years old and the leading cause of child undernutrition (1). Nearly all of these preventable deaths occur in low- and middle-income countries (LMICs) and would be prevented with effective case management of diarrhea (2). In 2004, the World Health Organization (WHO) and the United Nations Children’s Fund (UNICEF) issued a joint statement adding zinc supplementation (ZS) to the recommended formula for low-osmolarity oral rehydration therapy (ORT) for case management of diarrhea. Zinc supplements given for 10 to 14 days during acute episodes of diarrhea can decrease the severity and duration of the episodes, as well as the recurrence of diarrhea in the next two to three months (3–5). However, implementation of ZS coupled with long-standing ORT protocols in LMICs has been weak (6).

Issues with previous ORT programming efforts have included: (a) lack of knowledge by parents on recognition of diarrhea and the signs and symptoms of severe dehydration, leading to delays in treatment-seeking (7, 8); (b) poor training and knowledge retention on the dosage and uses of ORT (9, 10); and (c) reliance on manufactured ORT products and poor availability of those products (11). Food-withholding in cases of diarrhea and increased stool output as a deterrent to ORT use have been widely reported (7, 12). In Guatemala, ineffective ORT training among both parents and health promoters has been a barrier to effective ORT uptake, in addition to the widespread belief that commercial ORT products carry greater health benefits than homemade solutions (11).

The inclusion of ZS with ORT in standard childhood diarrheal treatment protocols offers hope for improving morbidity and mortality outcomes in Guatemala, where diarrhea is the third leading cause of death in children under 5 years old and an estimated 70% of children face chronic undernutrition (13). Low household economic status and indigenous cultural beliefs about anticipated episodes of diarrhea at certain milestones of child development are known to delay treatment-seeking for childhood diarrhea in Guatemala (7, 8, 14). Despite ample clinical evidence in support of ZS and potential for high acceptability by target populations, the new protocols have not yet been widely field-tested or adequately implemented in many LMICs. The Guatemalan Ministry of Health announced the implementation of ZS with ORT protocols and the funding of 4 million doses of zinc in 2011 (15); however, the diffusion of ZS as an addition to Guatemala’s national ORT protocols has not been systematically evaluated.

The objectives of this study were to: (1) co-design an ORT/ZS training program with community health promoters that is appropriate to the local context in a rural indigenous Guatemalan community and (2) understand how implementation of this training changed attitudes and behaviors of participating community members.

## MATERIALS AND METHODS

This observational study was conducted in a rural Mayan community with a population of approximately 15 000 in Guatemala’s highland department of Sololá. The community is spread across mountains and ravines surrounding a small central district in the valley floor that has local government offices, a church, and a few shops. The city of Sololá is the department capital (approximately 45 minutes away from the study community by bus), and it has the nearest health facility staffed by a doctor. Formal health services in the rural Mayan study community consist of a health post in the central district staffed by one nurse and a small team of assistants, plus four community centers in the furthest districts. The community has two pharmacies and several privately owned shops that stock a variety of medicines, largely analgesics, antibiotics, and antimicrobials.

Field activities for this study were conducted between January 2013 and June 2014. In January 2013, in-depth interviews were conducted with the mayor, health post staff, local pharmacists, the director of health services for the municipality (who is in the city of Sololá), the head of the pediatric unit at the departmental hospital (also in the city of Sololá), and existing health promoters (*n* = 8). Existing health promoters in the study community included both governmentcontracted and private, fee-for-service individuals who dispensed injections and medicines and provided basic health advice. Baseline data showed that community members had difficulty differentiating government promoters, whose services should be free, from private promoters. The existing promoters in the community at the time of the study were all males except for one individual from outside the community who worked for an area NGO. Four focus-group discussions were held with local women (*n* = 25), and 24 household surveys were conducted to understand local perceptions of childhood diarrhea, its prevalence in the community, and normative ideas about the types and sources of treatments used for childhood diarrhea.

This baseline community information was used to develop an interactive curriculum of five half-day sessions to train community health promoters. The curriculum incorporated local cultural norms and resources along with the WHO ORT/ZS guidelines on symptoms and severity of dehydration, differentiation of types of diarrhea, homemade oral rehydration solution (ORS), and dosages of zinc (16). The promoters were also trained in principles of adult education and how to work as health promoters in their community. Twenty women, selected by local leaders, attended the fiveday training session in June 2013. None of the women had previously worked as a health promoter in their community, a role that had typically gone to men. During the training, participants worked with the study team to co-design the curriculum that they would in turn share with community members, continuing the process of adapting WHO guidelines to the local context and developing a plan for when and how the information could best be shared within the community.

Seven of the 20 women met the study criteria of a post-training knowledge assessment, using both demonstration, pictorial, and written methods, and became volunteer community health promoters. The seven study promoters then began conducting trainings in other households in the community, and they were also available for advice through consultations during bouts of childhood diarrhea. Consultations were typically sought by community members who had previously received training from a study promoter, though a few untrained community members sought their help after receiving a word-of-mouth recommendation. The study promoters volunteered their time but were incentivized with mobile phone airtime added to their phones monthly if they remained active in conducting trainings or consultations.

Over the 12 months following the training, from June 2013 to June 2014, the seven study promoters reported giving training to or being consulted by 55 local women. Two of the study promoters were selected by the study team to act as group leaders, who would organize group meetings and share information with the others. The first author communicated with these leaders on a monthly basis by phone call and SMS (text message) to encourage continued participation and answer questions about training materials. In the final phase of the study, a household survey (*n* = 21) was conducted with women who had received training from the study promoters. The municipal health director in the department capital of Sololá and the local health post nurse in the study community were interviewed (*n* = 2). Finally, in-depth interviews and a group discussion were held with six study promoters who completed the project.

Ethical approval for this study came from the institutional review boards of Clemson University (United States) and Agnes Scott College (United States), in addition to local approval from the ethics board of Wuqu’ Kawoq, a Guatemalan health provision NGO respected in the region, and from the town council of the study location. Informed consent was obtained from all study participants, and community health promoters received an adapted version of the U.S. National Institutes of Health human subjects protection training. All personal identifiers were removed during data entry to a secure database, and written materials were destroyed to protect confidentiality of participants. All quantitative data were analyzed using MATLAB (Version R2014a), and qualitative data were coded and analyzed using QSR NVivo 10 software.

## RESULTS

### Community level

Diarrhea was widely recognized in the community as a serious problem for children (Table 1). During baseline community assessment, one focus group participant stated that diarrhea “is a common illness, but it is dangerous and can kill if there’s no way of treating it” (Focus Group Discussion 4). Ideas about treatment for childhood diarrhea centered largely on medicines, primarily tablets, at baseline. Availability of money was the most-mentioned determinant of where health care is sought, with a preference for pharmaceuticals or paid trips to doctors for most conditions that are not resolved with home treatment. The community’s mayor observed, “The economics make it difficult [for treatmentseeking]. Also, the distance to [the department capital of] Sololá [to the free government health center or hospital] is a problem—people have to pay to get there.” However, the more expensive bottled solutions were favored over the dry packets given free through the health post or at low cost in shops. One focus group participant stated, “We buy it in packets, make it with boiled water, and sometimes buy it in liquid [bottled]…the children prefer the bottled. They don’t like the packets because it tastes salty” (Focus Group Discussion 2).

**TABLE 1.** Rural indigenous community members’ knowledge of diarrhea, oral rehydration therapy, and zinc before and after training by local promoters, Guatemala, 2013–2014^a^

	Community baseline, January 2013 (*n* = 24)	Community members who had received training with a study promoter, as of March 2014 (*n* = 21)
Percentage who say children in their community suffer from diarrhea	88%	95%
Most common treatments for diarrhea in their community (listed in order of frequency; some respondents gave more than one answer)	Medicines (*n* = 12) Herbs/natural remedies (*n* = 9) Health professional (*n* = 4) ORT (*n* = 3)	ORT (*n* = 15)^b^ Medicines (*n* = 7) Health professional (*n* = 6) Herbs/natural remedies (*n* = 5)
Percentage who have heard of ORT	79%	100%^b^
Percentage who have used ORT	63%	95%^b^
Type of ORT used (listed in order of frequency; some respondents gave more than one answer)	Dry packets (*n* = 14) Bottled (*n* = 7) Homemade (*n* = 0)	Homemade (*n* = 11)^b^ Dry packets (*n* = 10) Bottled (*n* = 5)
Percentage who have heard of zinc as treatment for childhood diarrhea	0%	24%^b^
Percentage who have used zinc for childhood diarrhea	0%	5%

a All data collected by the authors during the study.

b Indicates a statistically significant increase (P<0.05), using Fisher’s exact test (25).

Although knowledge of ORT at baseline was 79%, only 63% reported having ever used ORT (Table 1). After training with a study promoter, both knowledge of ORT and actual reported use of ORT increased significantly. Following training from the study promoters, the trained community members most often cited homemade ORS as the most frequent type of ORT they used. They often expressed surprise and satisfaction with the knowledge that they could make an effective ORS at home for very low cost and without the inconvenience of a trip to the health post or pharmacy.

The percentage of community members who had heard of ZS did increase significantly after training with a study promoter, building on a baseline of 0%. Use of zinc for childhood diarrhea did not increase significantly, because zinc was unavailable through the health post or local pharmacies during the study period. The study promoters voiced frustration about lack of zinc availability in the community. They also reported that community members became confused when zinc was recommended as a treatment for diarrhea during their training sessions because the study promoters had to simultaneously tell participants that zinc was unavailable. Many of the study promoters reported they had begun to exclude the information about zinc from their training sessions with community members by the end of the study period because of these factors.

At baseline, the role of “health promoters” was found to be unclear among community members, with some promoters operating as a part of the government health service and others working as private providers. Although government services are supposed to be free, community members most frequently reported that the help of a promoter (public or private) was expensive.

Community members who received training from a study promoter reported significant increases in having consulted a health promoter and in holding the opinion that health promoters offer good assistance in times of illness (Table 2). The women who participated as health promoters reported feelings of self-efficacy and pride in their roles. One stated, “I feel useful to society and satisfied knowing that people practice it [the ORT education she gives]” (Promoter Follow-up Interview 1).

### Health system level

During the 18 months of field implementation of the project, zinc was never available in the research community through the government health post or private pharmacies and shops. The study promoters reported that the nurse in charge of the village health post told people that they “don’t have the right” to ask for specific medicines, such as zinc, and that the long-term stockout was beyond local control. That is despite the fact that traveling to the municipal health center in the department capital of Sololáto pick up supplies of medications and transporting them back to the post is an assigned duty for the health post nurse.

**TABLE 2.** Rural indigenous community members’ knowledge and opinion of community health promoters before and after training by local health promoters, Guatemala, 2013–2014^a^

	Community baseline, January 2013 (*n* = 24)	Community members who had received training with a study promoter, as of March 2014 (*n* = 21)
Percentage who say there is a health promoter in their community	79%	86%
Percentage who say they have consulted a health promoter for help with an illness	58%	90%^b^
Percentage who say health promoters offer good assistance for people who are ill	63%	90%^b^

a All data collected by the authors during the study.

b Indicates a statistically significant increase (P<0.05), using Fisher’s exact test (25).

The job of health post nurse changed hands in the final six months of the study, and the new nurse stated a desire to make changes in how the post had been run. Some community members reported feeling that the nature of a salaried job in the post made personnel lazy, while others felt the post was an important health resource in the community. At the conclusion of the study, three of the women trained as study promoters explored a continuing relationship with the health post as a way to reach more families about ORT/ZS in the community. The first author facilitated a meeting between the study promoters and the health post nurse, during which the women described their work as volunteer promoters. The health post nurse expressed interest in having them continue to conduct trainings and to coordinate with other community trainings carried out by health post staff. The women tentatively agreed to continue but said it would depend on the time commitment required and the consent of their husbands and fathers; they all felt it would be difficult to volunteer significant amounts of time on an ongoing basis.

Zinc was typically available at the municipal health center in the department capital of Sololá, the primary point of referral from the community health post. The municipal health center has a rehydration unit where children with diarrhea can have ORT administered. The nurses who operate the rehydration unit reported being out of zinc for two full months in 2012 and having intermittent shorter periods of stockouts in 2013. Stockouts were also reported at the departmental hospital in the city of Sololá, where many local community members reported seeking care when cases of childhood diarrhea became severe or when services were unavailable at the local health post. No private pharmacies in either the nearest town with bus connections or the department capital had zinc in tablet form that would meet the WHO guidelines on dosage. Some pharmacies had zinc in syrup format, but none said they would recommend it for a child with diarrhea.

The municipal health center in the city of Sololá is the administrative unit that oversees the operation of local-level health posts. Health center employees reported that the health post in the study community had been out of zinc for nine months during 2012–2013. The nonindigenous director of the municipal health center stated a belief that the biggest challenge in providing health care is the culture [of rural, indigenous populations], because “people only want a tablet and a cure. They don’t like prevention. Health is only a focus in [times of] illness.” Further, he said that for the rural population, such as in the study community, “it’s normal for a child to have diarrhea.”

## DISCUSSION

Rates of ORT knowledge and reported use increased in the study community. Even in contexts where comprehensive ORT and ZS implementation efforts have been made, behavior change according to the 2004 WHO/UNICEF protocol has been difficult to achieve (9, 17, 18). A previous study conducted in Guatemala demonstrated increased ORT/ZS knowledge of community health workers, but it was unclear if the training was effectively disseminated into the community and how parental behaviors related to ORT and ZS were influenced by the training (19). Our study demonstrates increased knowledge of ORT and ZS among community members trained by the study promoters. Training on homemade ORT increased both accessibility and affordability for community members who otherwise would have to undertake a trip to a government health facility or pay for ORT in a pharmacy or shop. Also, since many community members mentioned use of home and natural remedies for diarrhea, homemade ORT was acceptable under local cultural norms and may help overcome delays in diarrheal treatment-seeking (7).

Parents in Guatemala frequently express concerns that ORT does not relieve diarrhea. Based on this belief, the parents choose not to use ORT, thus indicating a lack of knowledge of the expected outcomes of ORT use (11). Co-administration of ORT and ZS, which shortens duration of diarrheal episodes and has growth benefits for nutritionally vulnerable children (20, 21), could carry the dual benefits of sustained ORT uptake while meeting parental expectations of effective treatment (22). A 2011 study found that in Guatemala approximately 30% of children who experience diarrhea are treated with ORT and 0% receive zinc for treatment of diarrhea (23). A subsequent study in 2013 found that the percentage of children treated with ORT was only 8% and the use of zinc remained low or nonexistent (8).

At the conclusion of our study, knowledge of ZS also remained low, and minimal usage was reported among community members trained by study promoters. Expressed desirability of ZS was high among both the study promoters and community members when they were made aware of its ability to shorten diarrheal episodes and prevent recurrence. However, we were unable to document significant uptake or satisfaction with ZS as intended since it was unavailable in the community during the study period. We believe health system staffing and zinc stockouts were the key barriers to household implementation of the WHO/UNICEF ORT/ZS protocol observed in this study, rather than sociocultural factors that were the presumptive cause according to upper-level Ministry of Health staff. Local health post staff and municipal level staff cited conflicting reasons for the local stockouts and, at times, denied them. ZS was adopted into the national formulary to come into line with WHO guidelines, but no changes in supply chain management were made to facilitate consistent delivery to the local level.

Previous interventions have used a community health promoter model for delivering health information in Guatemala, with positive results (24). The training of local women as health promoters was well accepted by the community, where promoters within the community had historically been primarily men. The model of using local women as promoters was met with satisfaction on both the part of the study promoters themselves as well as the female community members they trained. Because women are the primary caregivers of children, female promoters focusing on childhood diarrhea were well accepted in the community and likely had greater opportunity to build rapport during in-home training sessions. Follow-up surveys of community members trained by the study promoters indicated that this rapport would yield confidence in asking for help from a promoter in future cases of illness. The study promoters reported some women whom they approached to participate in training responded with suspicion because they were concerned that the promoters were seeking to financially benefit from them, likely due to the history of health promoters expecting payment in the community. Our study promoters demonstrated a willingness to undertake their role as a voluntary form of social participation in the community, supported by the significant shift seen in opinions on the quality of assistance offered by health promoters during the study. However, long-term work as volunteer promoters may be difficult to maintain, as indicated by the study promoters’ responses to the meeting with the health post nurse regarding continuation of their training activities.

Limitations of our study include the small sample size of promoters and of households trained by promoters during the study. Also, the study was unable to achieve its objective of measuring attitudes toward and having significant uptake of ZS because it was never available in the study community.

Translating a global protocol such as ORT/ZS into reality at the local level presents important challenges. At the community level, these challenges include creating acceptable health-seeking options for end-users that are simultaneously achievable for local implementation teams. At the health system level, among the difficulties are institutionalizing availability and accessibility of the recommended treatment. This example of collaborative work with the study promoters shows that the WHO/UNICEF guidelines could be adapted to reflect the experiences and resources in their community. As a result, the participating community members were satisfied and confident in the ORT training they received from the study promoters. Our female study promoters were able to build rapport with the local women, who were their friends and neighbors and who participated in their training sessions even though those women did not view other health workers as being accessible. While the local adaptations of ORT/ZS guidelines will vary depending on context, this study illustrates potential gains in accessibility, acceptability, and affordability for LMICs implementing ORT/ZS programs. We recommend a process of baseline data collection on knowledge and use of ORT/ZS, co-design of training with local input to create local ownership, and ongoing support of community health promoters. The successful adaptation of WHO protocols to local contexts also relies upon the commitment and ability of the health system to supply the necessary resources and to accept accountability for implementation.

### Funding.

Research reported in this publication was supported by the National Institute of Nursing Research of the National Institutes of Health under Award Number RO3NR013228. The content is solely the responsibility of the authors and does not necessarily represent the official views of the National Institutes of Health.

### Disclaimer.

Authors hold sole responsibility for the views expressed in the manuscript, which may not necessarily reflect the opinion or policy of the *RPSP/PAJPH* or PAHO.
